# Dynamical characteristics of AC-driven hybrid WSe_2_ monolayer/AlGaInP quantum wells light-emitting device

**DOI:** 10.1186/s11671-023-03920-7

**Published:** 2023-11-09

**Authors:** James Singh Konthoujam, Yen-Shou Lin, Ya-Hui Chang, Hsiang-Ting Lin, Chiao-Yun Chang, Yu-Wei Zhang, Shih-Yen Lin, Hao-Chung Kuo, Min-Hsiung Shih

**Affiliations:** 1 Research Center for Applied Sciences (RCAS), Academia Sinica, Taipei, 11529 Taiwan; 2 Department of Photonics and Institute of Electro-Optical Engineering, National Yang Ming Chiao Tung University, Hsinchu, 30010 Taiwan; 3 Graduate Institute of Electronics Engineering, National Taiwan University, Taipei, 10617 Taiwan; 4 Department of Photonics, National Sun Yat-Sen University, Kaohsiung, 80424 Taiwan

## Abstract

The exploration of functional light-emitting devices and numerous optoelectronic applications can be accomplished on an elegant platform provided by rapidly developing transition metal dichalcogenides (TMDCs). However, TMDCs-based light emitting devices encounter certain serious difficulties, such as high resistance losses from ohmic contacts or the need for complex heterostructures, which restricts the device applications. Despite the fact that AC-driven light emitting devices have developed ways to overcome these challenges, there is still a significant demand for multiple wavelength emission from a single device, which is necessary for full color light emitting devices. Here, we developed a dual-color AC-driven light-emitting device by integrating the WSe_2_ monolayer and AlGaInP–GaInP multiple quantum well (MQW) structures in the form of capacitor structure using AlOx insulating layer between the two emitters. In order to comprehend the characteristics of the hybrid device under various driving circumstances, we investigate the frequency-dependent EL intensity of the hybrid device using an equivalent RC circuit model. The time-resolved electroluminescence (TREL) characteristics of the hybrid device were analyzed in details to elucidate the underlying physical mechanisms governing its performance under varying applied frequencies. This dual-color hybrid light-emitting device enables the use of 2-D TMDC-based light emitters in a wider range of applications, including broad-band LEDs, quantum display systems, and chip-scale optoelectronic integrated systems.

**Graphical Abstract:**

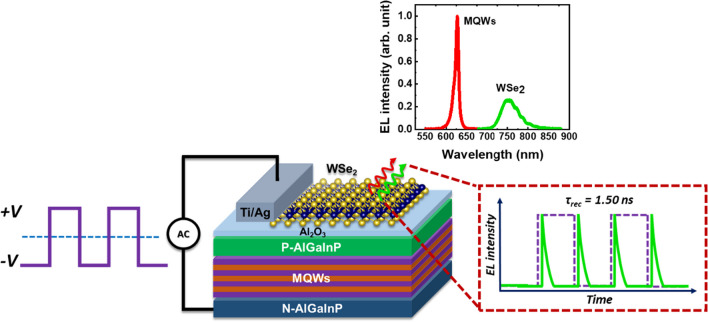

## Introduction

Monolayer transition metal dichalcogenides (TMDCs) are a class of direct bandgap two-dimensional (2D) semiconducting materials that show considerable potential for next-generation optoelectronic devices such as light emitting diodes (LEDs) and photodetectors [1–9]. Because high quantum efficiency may be achieved by utilizing direct band gap material as the emitter, their special properties, such as layer dependent bandgap and large exciton binding energy, make them a promising contender for light emitting applications. The absence of surface dangling bonds in TMDC atomic layers enables its integration with a three-dimensional (3D) bulk material to produce 2D/3D heterojunction devices, which will be crucial for various optoelectronic applications [10–13]. TMDC-based light emitting devices have been demonstrated in a variety of architectures, as either p-n junctions or capacitors depending on the driving source [5, 14–18]. LEDs based on TMDCs have demonstrated remarkable electroluminescence (EL) quantum efficiencies in the past few decades, with values ranging from 1 to 5% under high vacuum conditions [1, 6, 12, 19]. However, the majority of practical applications necessitate operation under ambient conditions, implying the need for diverse light emitting devices with high performance in such circumstances. In addition, large area TMDCs-based light emitting devices exhibiting high photoluminescence quantum yield (PLQY) have also been achieved due to recent developments in the synthesis of high quality TMDCs using chemical vapor deposition [20, 21]. The large resistive losses caused by the ohmic contact established between the metal and TMDC interfaces in the p–n junction structure, on the other hand, constitute one of the biggest obstacles for TMDCs-based light emitting devices [22, 23]. Several techniques, including the choice of an appropriate contact metal, doping, the introduction of a spacer layer at the interface, and the use of contact geometry, can reduce this contact resistance; nevertheless, these techniques complicate the manufacturing processes [24–28]. As a result, the lack of a suitable ohmic contact remains the ultimate challenge thereby limiting the performance of TMDC-based light emitting devices.

Alternating current (AC)-driven EL devices offer significant advantages when compared to traditional DC-driven light-emitting diodes in terms of power efficiency, charge accumulation, the ability of the dielectric spacer to inhibit electrochemical reactions from charge injection, and operational lifetime [29, 30]. These AC-driven EL devices are composed of emissive and insulating layers, which can exhibit tunable emission wavelengths, enabling their potential for a variety of optoelectronic applications. The inclination toward nanoscale miniaturization in electronics and photonics, which demands for nanoscale and high resolution light emitting sources, has resulted in the development of AC-driven devices based on single layer TMDCs. Despite the success of TMDC-based AC-driven devices [4, 5, 31], there is still a high demand for multiple wavelength emission from a single device, which is essential for full color light emitting devices. Additionally, optimization of the device structure is required to make the emission wavelengths from the devices more compatible when integrated with other light emitters. TMDC-based AC-driven EL devices are typically fabricated with capacitor structure using an insulating spacer between the emission layer and the substrate. Most importantly, as the charge injection process is nearly independent of the Schottky barrier's height under the application of AC pulsed voltage, an efficient carrier injection can be achieved at the metal-TMDC interface [32]. The TMDC-based light-emitting capacitor has two terminals, the source of which is grounded, and the gate electrode is connected to an AC voltage. By injecting electrons and holes independently, this capacitor configuration will generate pulsed light as a result of the electron charging and discharging effect, allowing researchers to study the EL emission pathway and carrier dynamics right down to the individual injection cycle [5, 33, 34]. Despite the potential of TMDCs in EL devices, the underlying mechanism governing how they function at various AC frequencies is still unclear. As a result, it is crucial to investigate these mechanisms in order to provide a more comprehensive understanding of TMDCs-based EL devices.

It has been successfully demonstrated that GaN-based LEDs, in addition to TMDC-based AC-driven EL devices, have vast development potential and wide-ranging application value in contemporary light-emitting technology [35–38]. Because of their spatial properties, which are driven by the demand for devices with features like small size, low power consumption, and ultrahigh resolution, nanoscale LEDs have drawn a lot of attention from the academic community. GaN-based nanoscale LEDs have been shown to enable emission wavelength tuning in the visible region by utilizing the same materials [39–45]. Therefore, it is possible to generate multiple light emission under the same circumstances by integrating TMDCs materials with the existing III-V-based light emitting technologies. Previous reports have shown that the EL intensities of GaN-based AC-LED have a linear dependence on the driving frequency [46]. The integration of TMDCs monolayer with an III-V epilayer opens the door to producing nanoscale LEDs capable of emitting multiple colors, due to incredibly thin nanoscale thickness of approximately 0.6 nm for TMDCs monolayer. As a result, TMDCs show great potential in advancing the development of next-generation nanoscale devices. Furthermore, TMDC-based AC-driven devices have been shown to exhibit frequency-dependent EL intensity and wavelength tunability, which is extremely important for multi-color devices [4, 5, 31]. While advanced optoelectronic applications can be made possible by wavelength-tunable LED devices, majority of the semiconductor LED devices that have been developed up to this point have a fixed emission wavelength. As a result, it is important to develop AC-driven devices having multiple emission wavelengths under the same operating conditions by controlling the driving frequency and device architecture. In this work, we demonstrated a two-color AC-driven light-emitting device with capacitor structure composed of a CVD-grown WSe_2_ monolayer transferred on top of an AlGaInP-based LED wafer to form a 2D/3D heterostructure. An AlO_x_ insulating layer is inserted between these two materials to serve as a capacitor layer. An equivalent RC circuit model is used to describe the underlying phenomenon on how the device's EL intensities are frequency dependent. This study provides a detailed understanding of TMDCs-based EL device under various driving circumstances by evaluating the transient electroluminescence response and the equivalent RC circuit.

## Results and Discussion

**Fig. 1:**
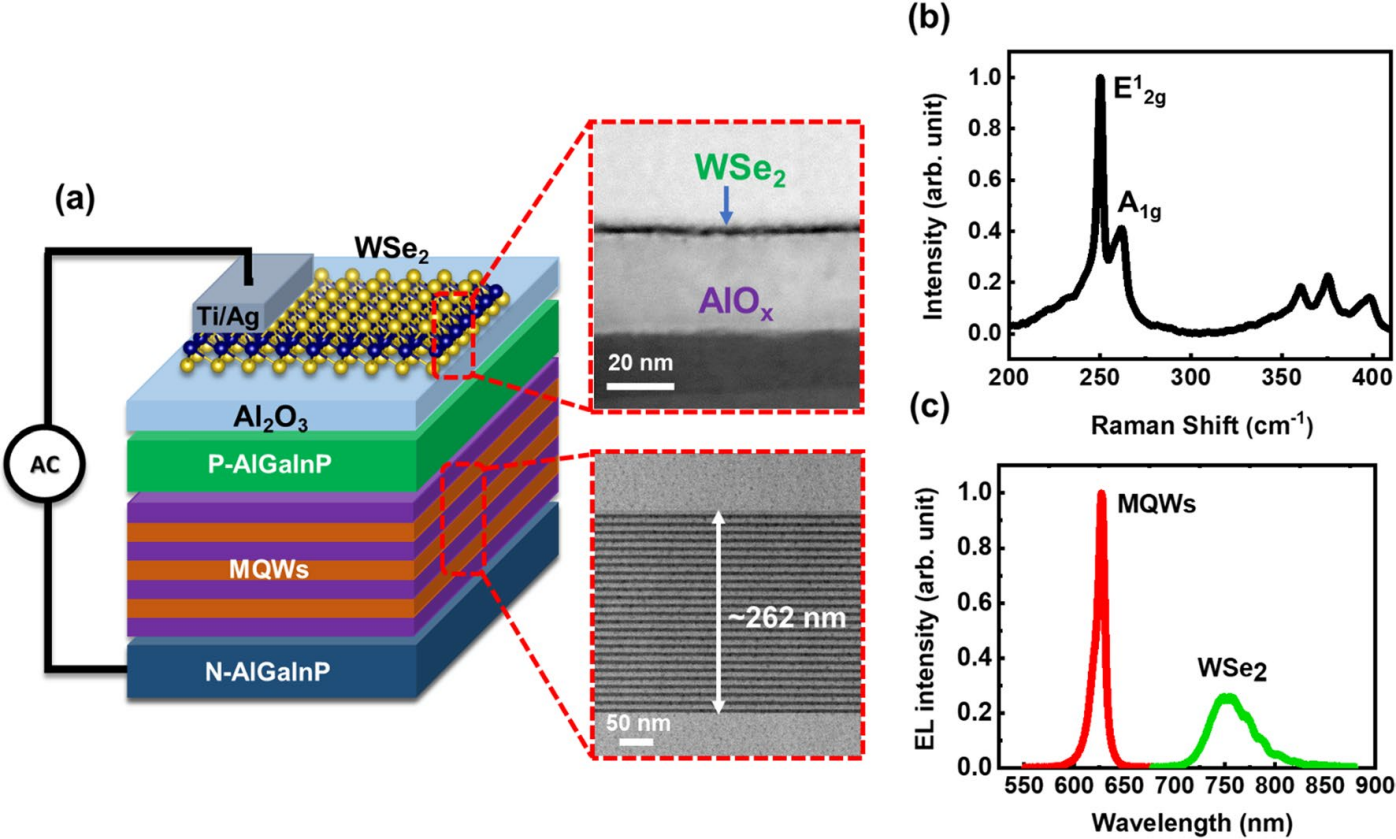
**a** Schematic of the hybrid WSe_2_ ML/GaP-based light-emitting device. Inset: Cross-sectional TEM images for the WSe_2_ monolayer and AlGaInP–GaInP MQWs. **b** Raman spectrum of the WSe_2_ monolayer on sapphire substrate. **c** EL spectrum of the hybrid device at fixed frequency (1 MHz) and voltage (12 V)

Figure 1a displays an illustrative depiction of the hybrid light-emitting device constructed with WSe_2_ monolayer and AlGaInP multiple quantum wells (MQWs). The device consists of a pair of silver electrodes, two active layers for emission i.e. AlGaInP-GaInP MQW and WSe_2_ monolayer and an insulating layer positioned between the two emitters. The electrode at the bottom serves as the gate while the one at the top serves as the source and the carrier injection takes place at the interface between the source contact and the WSe_2_ active layer. An insulating spacer with a high dielectric constant, comprised of a layer of AlOx film, is selected, which will lead to the generation of an internal electric field within the device. The AlO_x_ layer is deposited in between the two emission layers i.e. WSe_2_ monolayer and AlGaInP-based LED epilayer, to function as a capacitive element. The WSe_2_ monolayer was transferred to the LED epilayer through liquid-phase exfoliation method. Silver electrodes are applied onto the upper surface of WSe_2_ monolayer, serving as the source electrode, and onto the exposed surface of the LED epilayer, functioning as the gate electrode. Due to the limited carrier diffusion length in TMDCs, the carriers are likely to radiatively recombine at the vicinity of the contact edge leading to optical emission. The inset of Fig. 1a presents the TEM images of the WSe_2_ monolayer and the GaInP–AlGaInP MQWs, respectively confirming their formation in the device structure. The thickness of the WSe_2_ monolayer and the MQWs were revealed from the TEM image to be approximately 0.7 nm and 262 nm respectively. Figure 1(b) clearly shows two separate peaks, labeled E^1^_2g_ and A_1g_, which are visible in the Raman spectra at 250.3 and 260 cm^−1^, respectively. The E^1^_2g_ peak belongs to the in-plane vibration of tungsten and sulfur atoms, while A_1g_ corresponds to the vibration of selenium atoms in the out-of-plane direction, indicating the formation of WSe_2_ monolayer. Figure 1c displays the room-temperature emission spectrum of the light-emitting device based on WSe_2_-AlGaInP operating at a fixed frequency. This spectrum exhibits two clearly distinct emission peaks, one at around 625 nm, attributed to the AlGaInP-based multiple quantum wells (MQWs), and the other at approximately 755 nm, associated with the WSe_2_ monolayer. These peaks are associated with the AlGaInP-based multiple quantum wells (MQWs) and the WSe_2_ monolayer, respectively. The oscillation pattern observed in the EL spectrum of the WSe_2_ emission may be due to an interference pattern from the AlGaInP-based LED. The intensity of the EL peak for the WSe_2_ monolayer has been enhanced by a factor of 10 to enhance visibility, compensating for its low emission efficiency. The red emission band exhibits an emission peak at 625 nm, characterized by a full width at half-maximum linewidth of 10 nm corresponding to the AlGaInP–InGaP MQWs. Meanwhile, the peak at a longer wavelength is attributed to the direct intralayer exciton emission from WSe_2_ monolayer, and this observation is consistent with previously reported findings [47, 48].

**Fig. 2:**
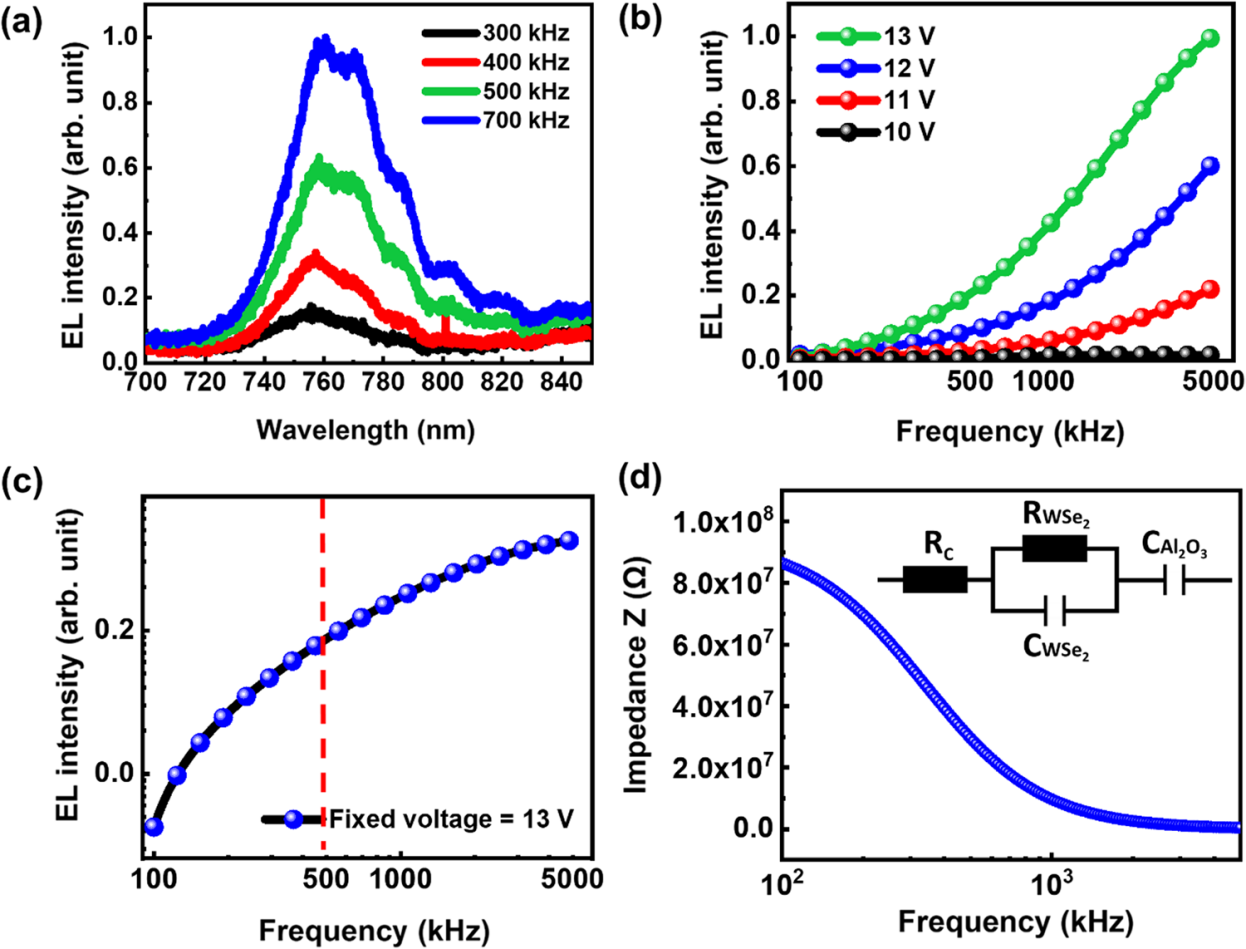
**a** Frequency dependent EL intensity of WSe_2_ at fixed voltage. **b** EL peak intensity of WSe_2_ operated by AC bias as the function of frequency under various applied voltage. **c** Semi-log plot of EL intensity-frequency. **d** Impedance of equivalent circuit with frequency sweeping from 1 Hz to 100 MHz

Figure 2a shows the room temperature EL spectra of WSe_2_ emission at various frequency under constant applied voltage of 12 V. The EL intensity of WSe_2_ emission increases with the frequency of AC voltage. Figure 2b shows the EL peak intensity of WSe_2_ operated by an AC bias as a function of frequency at fixed gate voltage and various applied voltage. The frequency-dependent EL peak intensity increases gradually with the frequency from 100 kHz to 5 MHz. The semi-log plot of EL intensity-frequency, as illustrated in Fig. 2c, reveals more information about the device features than the EL intensity-frequency curves given in Fig. 2b. We clearly observed a rapid increase of EL intensity as the frequency is sweeping from 100 to 500 kHz, indicating that EL intensity has a large sensitivity to the change in frequency in this region. After reaching the knee point of frequency, the rate of increase for the EL intensity gradually slows down. There are two likely explanations for this phenomena—the first one is that the number of transient pulses increases per unit time due to rising the frequency of the square-wave signal. As a result, a greater number of carriers are injected into the WSe_2_ per unit time that contributes to an enhancement in light emission. The relation of current density and frequency can be expressed as: $\mathrm{j}={\upepsilon }_{\mathrm{I}}\left({\mathrm{E}}_{\rm{I}}-{\mathrm{E}}_{\rm{B}}\right)\mathrm{f}$, here j is the current density passing through the electrode/WSe_2_ interface and f is the frequency of the square wave AC signal [49, 50]. The other explanation is described by a simplified equivalent RC circuit model consisting of resistors and capacitors connected in parallel and in series with a resistor as shown in the inset of Fig. 2d.

The AC-driven light emitting device with a single dielectric spacer is identical to a resistance–capacitance circuit from the standpoint of circuit analysis. The vector sum of resistance and capacitive reactance can be considered constant at a fixed frequency. In an AC circuit, reactance behaves similarly to resistance but is affected by supply frequency, unlike resistance, which is frequency-independent. When a direct current (DC) voltage is introduced into an RC circuit, the capacitor begins to accumulate a charging current from the power source, gradually charging up until it attains a level equivalent to the applied voltage. However, in the context of an AC circuit, the rate at which the applied voltage signal oscillates between positive and negative polarity is governed by the frequency of the supply. The frequency of the power supply determines how quickly the capacitor undergoes a continuous cycle of charging and discharging, leading to the flow of current through it, which is constrained by the capacitive reactance. When the frequency applied to the capacitor increases, the capacitive reactance decreases and vice versa and this variation can be referred to as complex impedance of the capacitor. The internal impedance of the capacitor seems to reduce with increasing frequency because, at higher frequencies, the capacitor allows a greater amount of charge to pass between its plates in a fixed time interval, resulting in a higher current flowing through it. Consequently, when a capacitor is connected to a circuit that fluctuates within a defined frequency range, it is referred to as being "frequency-dependent". Further details will be explained in the later section regarding frequency dependency of resistance and capacitive reactance.

By examining the variation in impedance as a function of the varying frequency (f) of the applied AC signal, we were able to evaluate the frequency-dependent impedance in an RC-equivalent circuit for our device in this section. Here, the resistors Rc, R_WSe2_, R_AlOx_, C_AlOx_, C_WSe2_ represent the contact resistance, sheet resistance of WSe_2_ and AlOx, the capacitance of AlOx and WSe_2_, respectively. As the result, we represent the impedance of the equivalent circuit by a complex number, Z (with the units of Ohm). The impedance Z is given by [51]:

1 $$ Z = R_{{\text{c}}} + \frac{1}{{\frac{1}{{R_{{{\text{AlOx}}}} }} + i{\upomega }C_{{{\text{AlOx}}}} }} + \frac{1}{{\frac{1}{{R_{{{\text{WSe2}}}} }} + i{\upomega }C_{{{\text{WSe2}}}} }} $$

where ω is the angular frequency, which is given by ω = 2πf. Impedance is a strongly frequency-dependent resistance on AC operating condition. The circuit's impedance varies in conjunction with the frequency variation in the AC signal. The capacitive reactance is large at low frequencies, resulting in a high overall impedance. As a result, the circuit will be less responsive to the applied AC signal and the light emission of the EL device will be dropped. The capacitive reactance drops at high frequencies, lowering the overall impedance, making the circuit more responsive to the applied AC signal, and increasing the light emission from the EL device. To further investigate the effect of frequency on impedance, we used LTspice to simulate the characteristic of frequency-dependent equivalent circuit model. Figure 2d shows the calculated impedance for the equivalent circuit with frequency sweeping from 1 Hz to 100 MHz. The impedance is relatively high at the low frequency region, then dramatically drops when the frequency is at ~ 500 kHz, which is consistent with the experimental data in Fig. 2b since the EL intensity starts to increase from 500 kHz. According to the above-mentioned equation, the impedance will be close to a constant value of R_0_ at ultrahigh frequency, so the impedance does not further decrease with continually increasing frequency. It can be observed that in the saturation region (frequency above 500 kHz), both the slopes of the simulated curve and experimental curve gradually flatten, which means the currents approaches saturation.

**Fig. 3:**
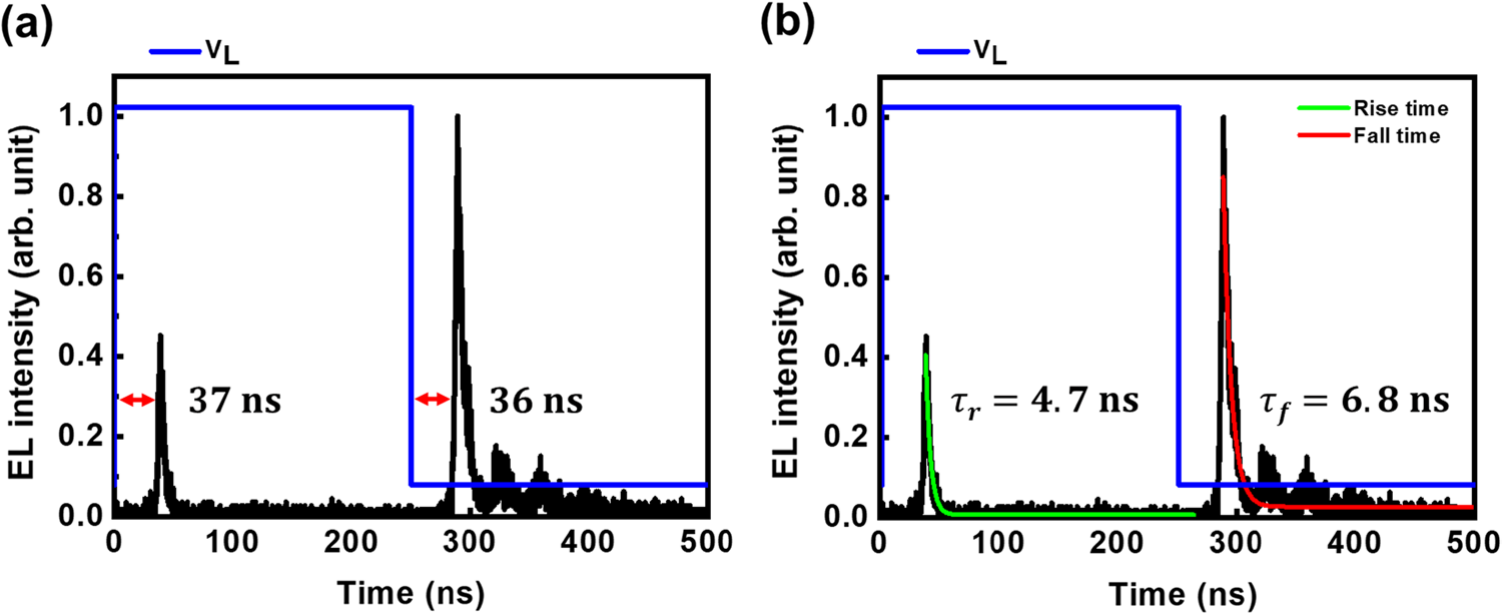
**a** Delayed emission during rise and fall time. **b** Decay characteristics of rise and fall region in TREL

The underlying physical mechanism of the device operation is further explained using the transient electroluminescence response to the applied voltage as given in Fig. 3. The pulse EL emission occurs at every transition of applied voltage, Vg and the transition has a full-width at half maximum (FWHM) of 5 ns. In a transient spanning from a positive gate voltage (+ V_g_) to a negative gate voltage (-V_g_), the number of electrons that can tunnel into the semiconductor remains minimal due to the substantial energy barrier presented by the Schottky barrier height for electrons. This leads to a reduced overall bipolar carrier concentration within the semiconductor, resulting in lower EL. Nonetheless, when transitioning from + V_g_ to − V_g_, a significant amount of holes is injected into the semiconductor due to the reduced barrier height. This leads to a net increase in the bipolar carrier concentration, subsequently resulting in a greater EL intensity. The device also exhibits a delayed emission characteristic, as shown in Fig. 3a, with a delayed time of 37 ns and 36 ns in respect to the rise and fall times, respectively. This behavior can be attributed to the accumulation and depletion of charges at the interfaces between the metal contact and WSe_2_ monolayer. Under the influence of the applied AC signal, charges accumulate at the interface during the positive half-cycle and deplete during the negative half-cycle. The emission of light takes place during the depletion phase after the accumulation cycle due to which the delayed emission that is visible in the TREL characteristics. The delayed times before the emission during rise and fall region for the device are approximately the same indicating the constant charge accumulation time and depletion time. However, these delay emission characteristics can be controlled by changing the frequency of the applied voltage signal, with which faster emission characteristics can be obtained. The decay time in rise time and fall time of the device EL characteristics is illustrated in Fig. 3b. It is clear that the decay time in TREL measurements is faster in the rise time i.e. 4.7 ns than in the fall time i.e. 6.8 ns. This is due to the fact that carriers are injected into the device and then recombine to produce EL during the rise time. The carrier recombination dynamics during the fall time, on the other hand, correspond to different charge trapping and non-recombination sites without carrier injections, resulting in a longer carrier decay lifetime. These nanosecond rise and fall times of this device will be very promising for modulation characteristics. This is because a faster carrier decay process allows the device to turn on or off more quickly, which will lead to more quickly transition between different states, resulting to faster modulation characteristics.

**Fig. 4:**
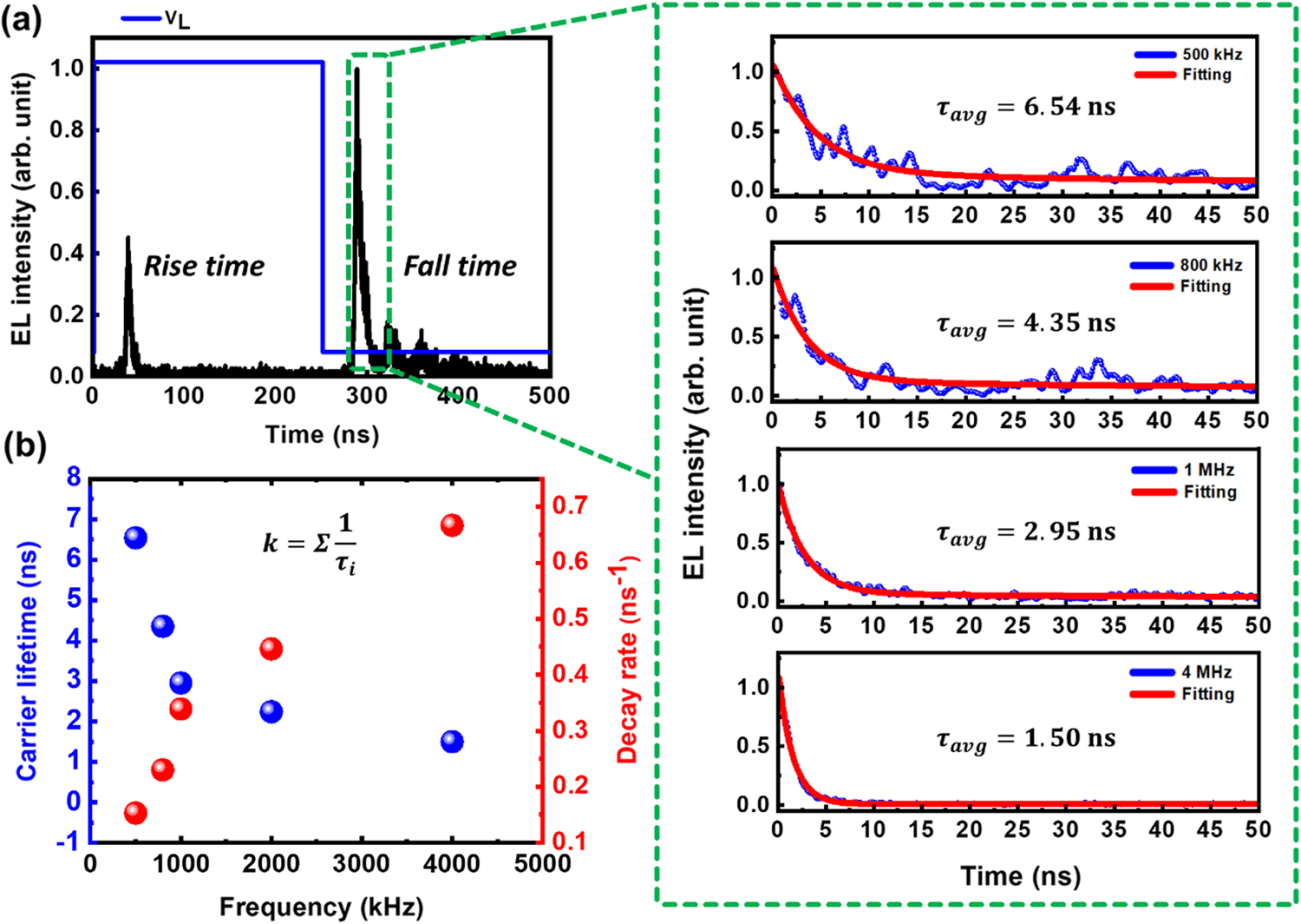
**a** Frequency dependent TREL (fall-time) characteristics of WSe_2_ emission at fixed applied voltage. The red line denotes the fitting curve and the decay time is given inside each figure. **b** Carrier lifetime and decay rates with respect to varying frequencies

We have further investigated the time-resolved electroluminescence (TREL) (fall-time) characteristics of the device in order to explain the underlying physical mechanism behind the frequency dependent EL. This decay curve oscillation could be attributed to the injection current oscillation that is generated from function generator [31]. The fall time can be used to estimate the active layer carrier lifespan, or the amount of time needed for the carriers to recombine for the EL intensity to fall. The frequency-dependent TREL in EL devices based on TMDCs can be explained in two distinct manners: by the recombination processes, which generate the EL emission, and by the carrier transport dynamics in the TMDC material. The TREL characteristics of the WSe_2_ monolayer emission in our device for different frequencies in shown in Fig. 4a. The FWHM for instrument response function (IRF) in our TRPL measurement system is 50 ps, which is significantly shorter than the measured decay time of the device. As a result, the IRF will have negligible influence on the measured decay time. The TREL curves are fitted monoexponentially and the carrier decay lifetimes are extracted for different frequencies, which are found to be 6.54, 4.35, 2.95, and 1.50 ns for 500 kHz, 800 kHz, 1 MHz, and 4 MHz respectively. It is clear that the carrier decay lifetime decreases with increase in frequency, which is attributed to a faster carrier recombination dynamics at high frequencies as shown in Fig. 4b. Higher frequencies typically result in faster electrical field oscillations, which enhances the amount of electron–hole pairs generated by the applied voltage. As a result, the exciton density in WSe_2_ monolayer increases, improving the probability of exciton recombination, thereby enhancing the rate of exciton recombination. The density of exciton traps can have an impact on the dynamics of carrier recombination in the WSe_2_ monolayer, which in turn affects the TREL decay lifetime. The presence of surface defects or impurities in WSe_2_ monolayer can act as traps for the charge carriers, leading to an increase in the recombination rate resulting in a faster decay of the EL signal at higher frequencies. The rise time also exhibits decay characteristics similar to that of fall time as we increase the frequency of the applied voltage. This is attributed to a faster radiative recombination dynamics owing to increase in exciton density at higher frequencies.

**Fig. 5:**
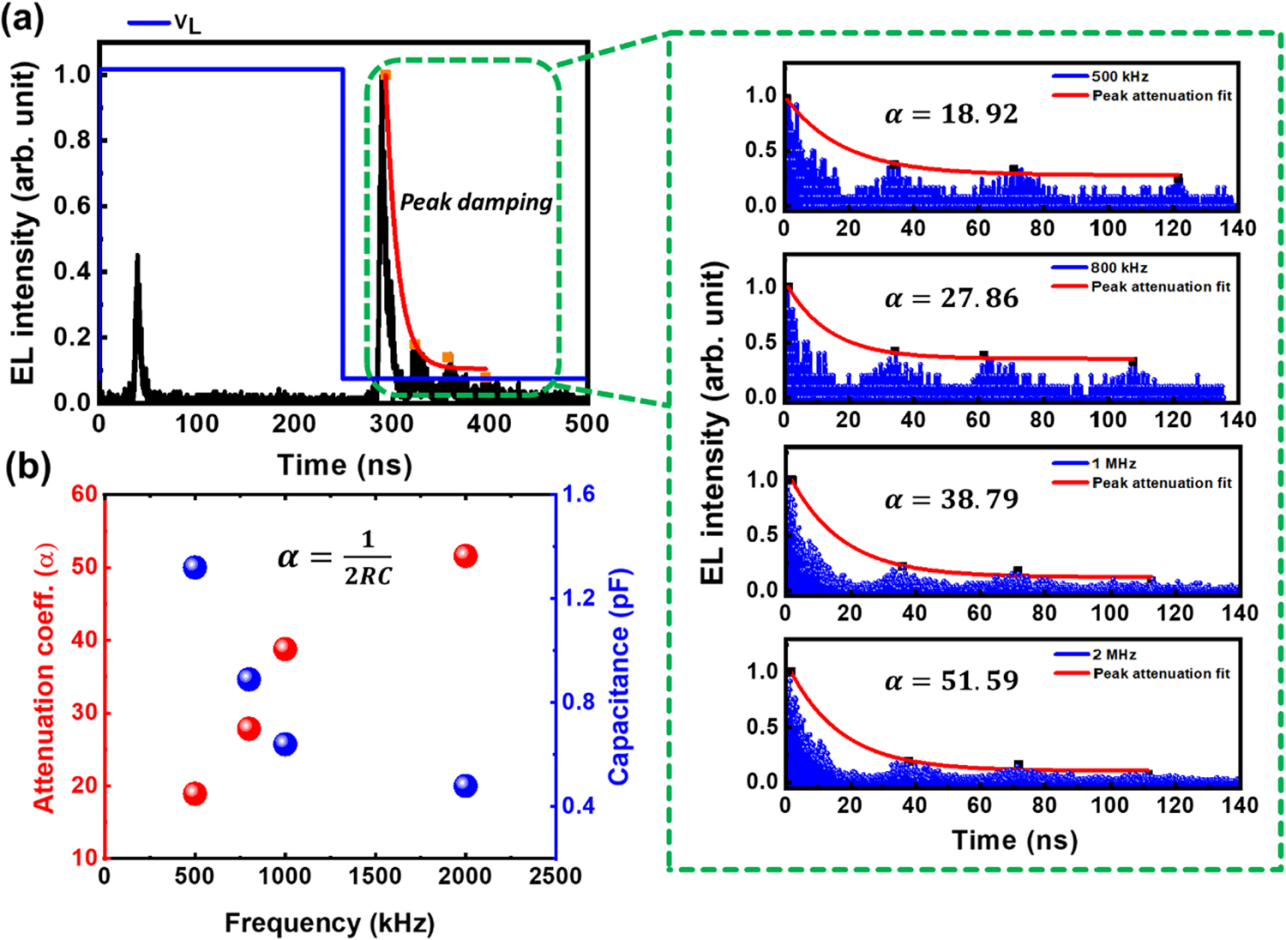
**a** Damping characteristics of TREL peaks. **b** Attenuation coefficients and capacitance for different frequencies

The frequency dependent EL characteristics is further explained using the attenuation coefficient (α) of the EL signal as shown in Fig. 5a. The EL oscillation damping may be attributed to the existing resistance and capacitance in the EL device, which can be further used to extract the attenuation coefficients. The attenuation coefficient is related to resistance and capacitance, which we considered in the RC circuit model as [52]

2 $$\alpha =\frac{1}{2RC}$$

where R is the resistance and C is the capacitance. The attenuation coefficients were estimated from the TREL curves to be 18.92, 27.86, 38.79, and 51.59 for 500 kHz, 800 kHz, 1 MHz, and 2 MHz respectively as shown in Fig. 5a.

The capacitance in the circuit model can be estimated by using the above relation after fixing the resistance in the circuit. The resistance is fixed at 20 GΩ as calculated using transmission line model (TLM) measurement. The estimated capacitance is found to be 1.32 × 10^–12^, 8.97 × 10^–13^, 6.44 × 10^–13^, and 4.84 × 10^–13^ F for 500 kHz, 800 kHz, 1 MHz, and 2 MHz respectively. The capacitance shows a decreasing trend with increase in frequency as shown in Fig. 5b. At low frequencies, the capacitance of the device is typically dominated by the interfacial capacitance between the device layers, which can be modeled using the parallel plate capacitor equation. However, as the frequencies increased, the rate of charging and discharging increased, resulting in a reduction in the capacitance value. Because of this faster charging-discharging rate, the EL intensity of the device increases with increasing frequencies. In addition to this, as the frequency of the AC signal increases, the capacitive reactance of the capacitor in the circuit model decreases, allowing more current to flow through the circuit. When an AC voltage is applied to the device, it causes electrons to move back and forth between the electrodes, leading to repeated cycles of excitation and relaxation. The increase in frequency leads to a decrease in the capacitive reactance of the device, allowing more current to flow through the circuit. This, in turn, leads to more electrons being excited and more photons being emitted, resulting to higher EL intensity. At low frequencies, charges have sufficient time to accumulate on the capacitor's plates, resulting in high capacitance. However, as the frequency increases, the time period for charge accumulation decreases, and the charges cannot fully accumulate on the active layer leading to a reduction in capacitance. However, there is an upper limit to the frequency at which the EL intensity can increase since the time for electron excitation and relaxation is limited. Therefore, increasing the frequency beyond a certain point may not result in a further increase in EL intensity.

## Conclusions

In this study, we demonstrated the influence of driving frequency on the carrier recombination dynamics of a dual-color AC-driven light emitting device constructed using WSe_2_ monolayer–AlGaInP MQWs. The EL peak intensity for both WSe_2_ and AlGaInP MQWs are found to increase with frequency, while the intensity attains saturation state at higher frequencies. This frequency dependent EL intensity has been explained well using an equivalent RC circuit model. The higher EL intensity of AlGaInP MQWs as compared to WSe_2_ monolayer is attributed to a high contact resistance at metal/WSe_2_ monolayer and faster impedance drop for AlGaInP. The frequency dependent characteristics of the device have been widely discussed using TREL and EL oscillation attenuation coefficient. The development of such dual-color light emitting devices based on TMDC monolayer opens up a wide range of 2D material applications and has the potential to result in the realization of various optoelectronic applications such as chip-scale integrated systems, broad spectrum LEDs, and quantum display systems.

## Methods

### Device fabrication

The WSe_2_-based device is created using a monolayer of WSe_2_ that is exfoliated from CVD-grown monolayers on the GaP substrate. The CVD growth process involved heating the Se powder to 260 °C and the WO_3_ powder to 925 °C, maintaining these temperatures throughout the process. Subsequently, the monolayer of WSe_2_, grown through CVD on a sapphire substrate, was transferred onto the GaP substrate using the liquid phase exfoliation technique. An insulating layer of aluminum oxide (Al_2_O_3_) was deposited between the substrate and WSe_2_ monolayer using atomic layer deposition (ALD) under a pressure of 10^–2^ Torr and at a temperature of 180 °C. Laser direct writer was employed to define the specific region for selective etching and electrode deposition in the monolayer WSe_2_ monolayer. The monolayer WSe_2_ was etched using Inductively Coupled Plasma (ICP), and the electrodes comprise of 5 nm titanium (Ti) and 150 nm silver (Ag).

### Characterization techniques

We utilized a home-built micro-PL setup to measure the optical and electrical characteristics of the device discussed in this study. In the micro-PL measurement, we employ a 450 nm continuous-wave (CW) laser to excite the device. The laser is focused using a 100 × objective lens with a numerical aperture (NA) of 0.55, resulting in a micrometer-scale spot size for the laser beam. Afterward, we employed a 532 nm dielectric long pass filter to obstruct the excitation light while permitting the PL signal to pass through. The PL signal underwent dispersion through a 300 grooves per mm grating and was subsequently detected using the Andor iDus 416 charge-coupled device (CCD) and the EL signal was measured using the identical setup. KEYSIGHT 33600A Waveform Generator is utilized to operate EL devices by applying it to the source contact, while the gate electrode remains grounded. PicoHarp 300 time-collected single-photon counting (TCSPC) module was utilized to gather the TREL measurements where the timing of TCSPC was synchronized by connecting the AC waveform generator trigger to ensure it matched the frequency and phase applied to the source contact. We used LTspice software to simulate the characteristic of frequency- dependent equivalent circuit model considered for the device.

## Data Availability

The data generated during the current study are available from the corresponding author on reasonable request.
